# A *Caenorhabditis *motif compendium for studying transcriptional gene regulation

**DOI:** 10.1186/1471-2164-9-30

**Published:** 2008-01-23

**Authors:** Christoph Dieterich, Ralf J Sommer

**Affiliations:** 1 Department of Evolutionary Biology, Max Planck Institute for Developmental Biology, Spemannstraße 35 - 37, Tübingen, Germany

## Abstract

**Background:**

Controlling gene expression is fundamental to biological complexity. The nematode *Caenorhabditis elegans *is an important model for studying principles of gene regulation in multi-cellular organisms. A comprehensive parts list of putative regulatory motifs was yet missing for this model system. In this study, we compile a set of putative regulatory motifs by combining evidence from conservation and expression data.

**Description:**

We present an unbiased comparative approach to a regulatory motif compendium for *Caenorhabditis *species. This involves the assembly of a new nematode genome, whole genome alignments and assessment of conserved *k-*mers counts. Candidate motifs are selected from a set of 9,500 randomly picked genes by three different motif discovery strategies. Motif candidates have to pass a conservation enrichment filter. Motif degeneracy and length are optimized. Retained motif descriptions are evaluated by expression data using a non-parametric test, which assesses expression changes due to the presence/absence of individual motifs. Finally, we also provide condition-specific motif ensembles by conditional tree analysis.

**Conclusion:**

The nematode genomes align surprisingly well despite high neutral substitution rates. Our pipeline delivers motif sets by three alternative strategies. Each set contains less than 400 motifs, which are significantly conserved and correlated with 214 out of 270 tested gene expression conditions. This motif compendium is an entry point to comprehensive studies on nematode gene regulation. The website: http://corg.eb.tuebingen.mpg.de/CMC has extensive query capabilities, supplements this article and supports the experimental list.

## Background

The era of whole genome sequencing has boosted functional analysis of eukaryotic genomes. Upon completion of model organism genomes like *Saccharomyces cerevisiae*, *Caenorhabditis elegans *and others, comparative sequencing has gradually moved into the sequencing focus. These sequencing efforts delivered and continue to deliver valuable insights into the evolution of function and species.

We are interested in transcriptional gene regulation exerted by genomic sequence and promoter regions in particular. Promoter regions play a crucial role in initiating transcription of a gene. Protein/DNA interactions regulate transcription initiation and confer specificity to this process. For a long time, yeast has been the primary model organism for research on eukaryotic gene regulation. From a bioinformatics perspective, gene regulation is far better understood in yeast than in any other eukaryote (e.g. [1]). Here, we consider the case of a multi-cellular organism, *Caenorhabditis elegans*. In this work, we compile a compendium of putative regulatory upstream elements by using sequence and functional genomics data (see website [2]). We define candidate motifs on conserved upstream regions of *C. elegans *genes as given in Wormbase 140. These candidate motifs are tested for their enrichment in conserved regions. This approach was previously pioneered for mammalian genomes [3] and yeast genomes ([4] and [5]). Subsequently, motifs are optimized with respect to length and specificity. Finally, motif candidates are evaluated based on the impact of motif's presence/absence pattern on gene expression as defined by experimental evidence (microarray data). The discriminative power of motif combinations is assessed with conditional trees.

### Species selection

*Caenorhabditis elegans *is a prime candidate for addressing questions of gene regulation in a multi-cellular setting. Most notably, its fixed cell lineage and thus defined number of cells render experiments comparable to the single cell level.

**Figure 1:**
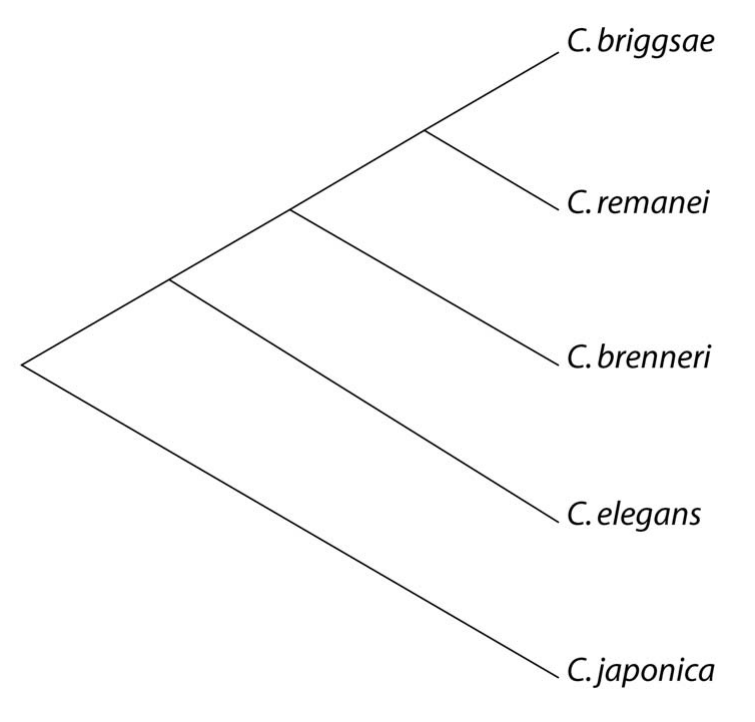
**Slanted cladogram of five *Caenorhabditis *species represented by living strains and corresponding whole genome projects**. The four top species form the *Elegans *group, which we consider in our analysis. This figure is adapted from [28].

Comparative approaches depend heavily on the available sequence data. Our goal is to create a compendium of short regulatory motifs (6 – 12 mers). This requires multiple alignments of nucleotide sequences. Recently, an initiative to sequence additional nematode genomes has gained momentum [6]. Genome sequencing of four species of the *Caenorhabditis *clade [7] (see Figure 1) is either completed (*Caenorhabditis elegans *and *Caenorhabditis briggsae*) or at an advanced stage (*Caenorhabditis remanei *and *Caenorhabditis brenneri*). We built our own assembly of the *Caenorhabditis remanei *and *Caenorhabditis brenneri *genome given the sufficient genome coverage (> 8-fold) of the ongoing sequencing projects.

To assess the suitability of the aforementioned species for phylogenetic footprinting, we estimated the neutral background substitution rate (*K*_*s*_) from synonymous substitutions in a multiple alignment of the RNAP2 gene (*ama-1*) [7]. Estimated values are 1.5029 for *C.elegans – C.remanei*, 1.7964 for *C. elegans – C. brenneri *and 2.2239 for *C.elegans – C.briggsae *using codeml [8]. Stein et al. [9] report similar values for the whole proteome comparison of *C.elegans – C.briggsae*. The molecular phylogeny based on a nucleotide sequence alignment of RNAP2 genes (*ama-1*) is in agreement with the one published by Kiontke et al. [7] (see Figure 1). They additionally used the SSU rRNA, the LSU rRNA as well as parts of the coding regions of *par-6 *and *pkc-3*. This phylogeny will guide us in building multiple alignments from pairwise ones. Intriguingly, the four *Caenorhabditis *genomes align pretty well despite the high estimates of the neutral background substitution rate (see Table 1). We first computed pairwise whole genome alignments of *C. elegans *and the other species. Subsequently, we merged pairwise alignments into a multiple alignment of all four species. Motif candidates are selected from multiple alignments whereas pairwise local alignments are retained for evaluating lineage specific motif abundance, which we will not discuss here. Future considerations will address issues like species-specific motifs and phylogenetic profiling of motifs in the satellite species *Pristionchus pacifcus *and distantly related species such as the human parasites *Brugia malayi *and *Trichinela spiralis*.

**Table 1:** Whole Genome Alignment coverage of the C. elegans genome

Species pair	Length	Coverage (%)
C. elegans – C. brenneri	39,781,786	~ 40%
C. elegans – C. remanei	40,670,546	~ 41%
C. elegans – C. briggsae	26,918,113	~ 27%
H. sapiens – M. musculus	-	~ 39% [14]

## Construction and content

### Genome assembly of *Caenorhabditis remanei *and *Caenorhabditis brenneri*

We downloaded a recent snapshot of the ongoing sequencing efforts from the NCBI trace archive [10]. We used the PCAP-REP assembler [11] to obtain a draft assembly for whole-genome alignment. Key features of the assemblies are median contig sizes of 17, 658 bp for *C. remanei *and 11, 912 bp for *C. brenneri *and median supercontig sizes of 202, 125 bp for *C. remanei *and 63, 873 bp for *C. brenneri*. Additional details are part of the Supplementary Materials. The preliminary assemblies were not manually refined and directly submitted to the following genome alignment step. The genome assemblies of *C. elegans *and *C. briggsae *were obtained from [12].

### Whole Genome Alignments

Pairwise comparisons of *C.elegans – C.briggsae *have been previously used for phylogenetic footprinting [13]. The two additional *Caenorhabditis *species are framed by this species pair in the molecular phylogeny we use (Figure 1). The whole set of four nematode genomes is consequently in an ideal range of sequence divergence for phylogenetic footprinting. This assumption is further supported by analyzing the alignments (see below).

We computed pairwise whole genome alignments of the *C. elegans *reference genome to the 3 other genomes. Pairwise whole genome alignments were computed using blastz [14] with default parameters except Y = 3400 and H = 2000. Multiple whole genome alignments were progressively built from pairwise alignments with multiz [15]: Sequences of *C. brenneri*, *C. remanei *and *C.briggsae *were merged to the *C.elegans *reference sequence in this order. Pairwise alignment coverage relative to *C. elegans *is given in Table 1. Alignment coverage of the *C. brenneri *or *C. remanei *to *C. elegans *is at a similar level as man-mouse comparisons.

*C.elegans *gene annotations from Wormbase release 140 [16] were projected onto the whole genome alignment to define upstream regions. Upstream sequences extend maximally over a range of 2 kb. If curated exonic sequence falls into that region, sequences are trimmed accordingly.

### Compilation of a motif compendium

We define motifs as strings composed of nucleotide IUPAC (International Union of Pure and Applied Chemistry) symbols, which contains atomic nucleotide symbols and redundant symbols.

**Figure 2:**
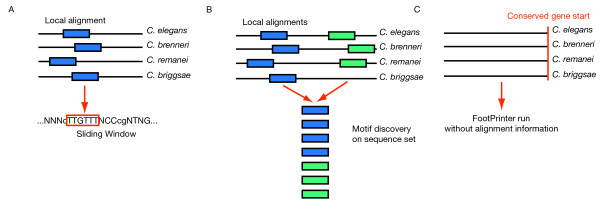
**Motif candidate compilation**. We employ three different strategies to extract motif candidates from genome sequences. A: Local alignments of 4 species are translated into IUPAC symbols. Only ungapped motifs (in capital letters) are collected with a sliding window approach. B: All subsequences that are covered by local alignments are collected and GEMODA is run on this file. C: FootPrinter is run on upstream regions where the gene start (first exon) is conserved in all four species.

To account for possible biases in motif discovery approaches, candidate motifs lists were generated from a set of 9,500 randomly selected upstream regions (almost 50% of all protein coding genes) with three different strategies (see Figure 2):

#### Strategy 1 – Kmers from 4-species local alignments

We collected all multiple alignments that contained at least four species and translated them into single IUPAC sequence representations using the alphabet ∑_*DNA' *_= {*A*, *C*, *G*, *T*, *N*} where N is a wildcard character, which represents any of the other characters (see Figure 2A). Alignment columns that contain gaps are translated into lower case letters whereas columns without gaps are translated into upper case letters. We collected all motifs of 6 to 12 base pair length from ungapped (upper case) alignment columns. Each motif could contain maximally two wildcard characters in total. Motif descriptions that start or end with two consecutive wildcard characters were excluded from the candidate set before the expression filtering step.

#### Strategy 2 – Motif discovery in local alignments

Motif candidates were sampled from upstream sequences that are covered by local alignments of at least two species (see Figure 2B). All conserved sequences of an individual sequence regions are subject to a motif discovery step using GEMODA. We used the following program parameters: -m dna_idmat, -l 6, -k 4, -g 5. GEMODA computes short multiple sequence alignments as motif descriptions in three distinct phases: comparison, clustering and convolution. During the comparison phase, short overlapping windows (6 mers) in the dataset are compared. During clustering, these windows are grouped together to form elementary motifs. We used the clique finding option to group motifs. Finally, during convolution, these motifs are stitched together to form maximal motifs. Further details are given in the original publication [17]. Motif candidates are retained if they have a P-value of < 0.05, a self-similarity of < 0.5 and a length of ≤ 12.

#### Strategy 3 – FootPrinter

The FootPrinter Motif Discovery software [18] does not use alignments as input. Instead, FootPrinter is run on homologous upstream regions. We consider upstream regions as homologous if they have a conserved gene start (first exon) in all four Canorhabditis species. FootPrinter uses a phylogenetic tree to evaluate the parsimony score of each potential motif. We used the tree shown in Figure 1. The Program parameters are set to default values except -sequence_type upstream, -subregion_size 100, -triple_filtering. All reported footprints are extracted per nematode sequence and clustered with GEMODA (same parameters as above) to yield a motif description.

Motif discovery parameters were selected in such a way that known motif description from Wormbook [19] meet these criteria.

We only consider motifs from 6 to 12 bp coming from these three discovery pipelines. Strategy 1 uses only multiple alignment across all four species (see Table 2 for the sequence space). Strategy 2 uses all available alignment information (pairwise and multiple alignments) whereas strategy 3 does not use any alignment information in the actual motif discovery process. Table 3 summarizes the different stages in the motif discovery process for each strategy.

**Table 2:** Detailed Alignment coverage for the set of 9500 randomly selected genes

No. Species	No. genes	Length of alignments
≥ 2	8,526	5,559,056 bp
≥ 3	5,026	1,796,951 bp
4	3,361	1,258,422 bp

**Table 3:** Conserved motif counts and motif processing

Conserved Motif counts			
Processing step	Kmer	GEMODA	FootPrinter

Initial candidates	404,546	256,688	41,747
Degeneracy optimization	193,491	82,672	24,247
Z-score and P-value	4,442	5,477	5,312
Expression data filter	Condition dependent (< 1,000)		

#### Motif conservation enrichment

Each motif library is tested separately for motif specific enrichment in conservation. Genomic upstream sequences from *C.elegans *constitute the motif background set. We scanned the respective upstream sequence alignments for conserved occurrences of candidate motifs. Alignment columns that contain gaps are not considered.

We employ a Z-score statistic to rank our motifs according to their enrichment in conserved regions.

(1) $$Z=\frac{x-np_{0}}{\sqrt{np_{0}(1-p_{0})}}$$

where *x *is the number of conserved instances of a motif minus the expected number of conserved instances divided by the standard deviation. The expected number of conserved motifs is the product of the number of occurences in genomic sequence (*n*) and the probability for a motif of being conserved (*p*_0_), which is the ratio of all conserved versus genomic occurences. P-values are computed for an exact test of the simple null hypothesis that *x *is *B*(*n*, *p*_0_) distributed. All motifs descriptions with a Z-score > 3 are retaine data 5% FDR level.

**Figure 3:**
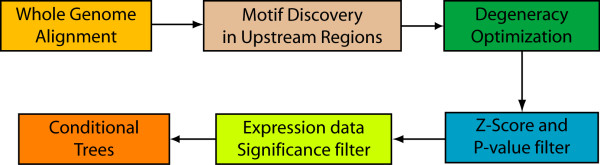
**Overview of motif extraction pipeline**. Schematic overview of motif processing steps. Gene structure annotations are projected across the whole genome alignments. Motif candidates are identified on a subset of 9,500 randomly picked upstream regions. Degenerate motif descriptions are removed if the set of atomic motifs, which they represent, scores better in terms of conservation enrichment. The greatest reduction in the number of candidate motifs is attained by scoring conservation (Z-Score and P-value filter with a 5% FDR level cutoff). Additionally, larger motifs are removed if smaller substrings (≥ 6 bp) of these motifs score better in terms of conservation. Motif candidates are then evaluated by a non-parametric test, which assesses their influence on gene expression. Finally, conditional trees are employed to select motif ensembles, which possibly have a joint regulatory function.

We prune the list of motif candidates by removing degenerate motifs based on their Z-score and P-values. This step halves the number of motif candidates (see Table 3). An overview of the entire processing pipeline is given in Figure 3.

#### Motif length selection

We further reduce our list of motif candidates by selecting for optimal motif length. Briefly, longer possibly degenerate motif descriptions are removed if a substring of the considered motif scores better in terms of Z-score and P-value. This step reduces the number of motif candidates to ~ 5,000 for each pipeline.

#### Motif significance filtering by expression profiles

We used a whole genome set of expression profiles for 270 conditions from Wormbase [16] to assess the individual importance of the presence of a motif on gene expression. We use the presence (copy number ≥ 1) or absence of a motif as indicator variable to split gene expression values for a particular condition into two sets.

**Figure 4:**
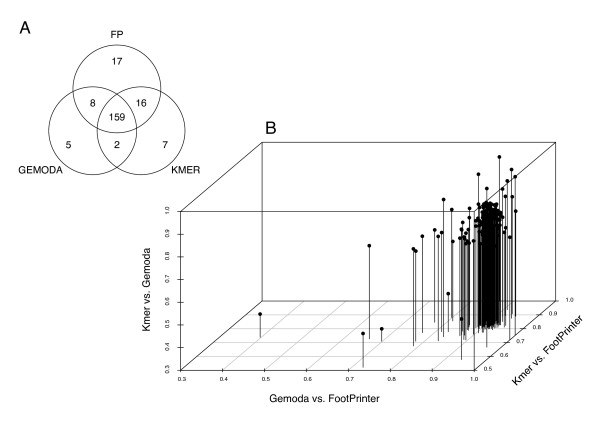
**Motif finder assessment**. **A: **We employ three different strategies to extract motif candidates from genome sequences. The statistical significance of a motif's presence has been tested on an expression data set containing 270 conditions. Motif sets have been reported by at least one approach for 214 conditions at a 5% FDR level. The distribution of the significant motif sets from all discovery pipelines is represented by the Venn diagram. **B: **Pairwise similarity comparison of motif sets from 159 expression conditions that are covered by predictions from all motif discovery pipelines. The scatterplot shows the distribution of 159 condition-specific average similarity values for each pairwise comparison of motif discovery strategies.

The two subsets are compared with the non-parametric, two-sample Wilcoxon rank sum test. Here, the null hypothesis states that the two distributions differ by a location shift of zero. We collect all motifs for which we could reject the null hypothesis at a 5% FDR level. The Venn diagram in Figure 4A summarizes the results for the three different motif discovery pipelines. In total, we could select significant motif candidate sets for 214 expression conditions by combining all three strategies. In essence, all strategies cover a large core set (n = 159) of gene expression conditions. However, a small set of 29 conditions is only covered by one of the three methods.

#### Motif set comparisons

We used an alignment approach to compare the motif descriptions from all three motif discovery pipelines on the large core set of expression conditions. Herein, pairwise motif set comparisons are carried out by alignment. Given two motif sets *A *= {*a*_1_, ..., *a*_*n*_} and *B *= {*b*_1_, ..., *b*_*m*_}. We select the smaller of the two sets: A if *n *<*m *or B else. We take the larger set as database *D *and perform all pairwise global alignments of the smaller set to *D*. Global motif alignments are computed with an implementation of the Needleman-Wunsch algorithm (EMBOSS program needle) and an extended DNA scoring scheme (Matrix NUC4.4 from [20]). Gap opening penalty is set to -10. Gap extension penalty is set to -0.5. The best matching pairs are retained. We normalize the scores according to this formula:

(2) $$\text{Score}\prime (a_{i},b_{j})=\frac{2\times \text{Score}(a_{i},b_{j})}{\text{Score}(a_{i},a_{i})+\text{Score}(b_{j},b_{j})}$$

with 1 ≤ *i *≤ *n *and 1 ≤ *j *≤ *m*. The mean score of the set of best scores is kept for each expression condition. The three-dimensional scatterplot in Figure 4B shows the distribution of average pairwise similarities of the motif predictions. The pairwise similarity of two condition-specific motif sets is expressed as the average of normalized best alignment scores (see above). Figure 4B indicates that condition-specific motif sets from different prediction pipelines show high similarities of ≥ 80% on average. In summary, the major share of our motif sets is found by three independent methods.

### Expression signature analysis by conditional trees

Conditional trees [21] were used to study the discriminatory power of our motif sets. The objective was to discover presence/absence pattern of several motifs that are significantly correlated with the expression level of a gene set. Significant split points support the hypothesis that a set of particular motifs influences the selected expression condition.

Mining for condition-specific motif patterns is effected with a recursive partitioning strategy. Only motifs that are conserved across all four species are taken into account. In other words, conditional trees estimate a regression relationship by binary recursive partitioning in a conditional inference framework [21]. In our case, conditional trees perform a regression over the motif counts as predictor variables.

The algorithm works as follows:

#### Conditional trees

1. Test the global null hypothesis of independence between any of the input variables and the response (presence or absence of a motif). Stop if this hypothesis cannot be rejected. Otherwise select the input variable with strongest association to the response. This association is measured by a P-value corresponding to a test for the partial null hypothesis of a single input variable and the response.

2. Implement a binary split in the selected input variable.

3. Recursively repeate steps 1) and 2).

We use the R implementation as in the party package (see [22] for details).

A high proportion of tested expression conditions (121 for the GEMODA strategy, 181 for the FootPrinter strategy and 171 for the Kmers strategy) shows significant associations with upstream motif patterns. All in all, we could assign 191 GEMODA motif descriptions, 255 Kmer motif descriptions and 340 FootPrinter motif descriptions to gene expression conditions by the conditional tree framework.

All conditional trees are deposited as Supplementary Material on [2].

### Utility

In our approach, sequence conservation is an indicator of functional relevance as many known examples of functional DNA motifs are under negative selection. This concept is also known as **phylogenetic footprinting **[23] and was successfully applied in the context of motif finding.

**Figure 5:**
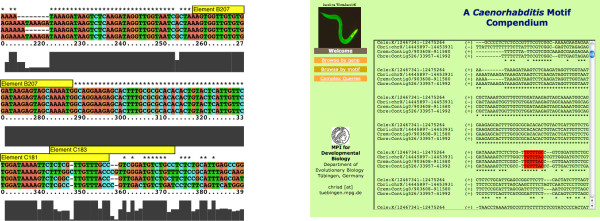
**Alignment of the myo-2 enhancer and corresponding web page view**. **Left: **Functional subelements of the *myo-2 *enhancer are highlighted by yellow boxes. The cell-type-specific subelement B207, which is identical in all species, binds and is activated by the pharyngeal muscle specific NK-2 family homeodomain factor CEH-22 [24] [29]. The organ-specific subelements C181 and C183 bind and are activated by the pan-pharyngeal FoxA family transcription factor PHA-4 [30], which is required for formation of pharyngeal muscle and all other pharyngeal cell types during embryonic development. The C elements are a little less conserved than B207, but the PHA-4 binding site matches the high-affinity consensus sequence TGTTTRC [31]. **Right: **Web page view of the same genomic region. The high-affinity consensus sequence TGTTTRC for PHA-4 binding is highlighted in red.

A closer look at the *myo-2 *enhancer, a well studied example of organ- and cell type-specific regulatory elements, demonstrates the utility of this approach. Figure 5 shows a schematic overview of the region in question and the corresponding display in our web service. The *myo-2 *enhancer is located ~ 300 bp upstream of the gene start. Transcriptional activity of *myo-2 *heavily depends on two elements B and C [24]. Okkema and Fire could pinpoint cell-specific and organ-specific activity to subelements (B207, C181 and C183) all of which are located in a small region of perfect sequence similarity among all four species. Nucleotide level views of multiple whole genome alignments of all four *Caenorhabditis *genomes are available via our accompanying web resource [2]. The web interface renders these alignments accessible either by scanning for a particular motif (browse by motif) or by studying a particular genomic loci (browse by gene) as shown with the *myo-2 *enhancer. A more coarse-grained view on motif occurrences is also provided via a GBrowse interface [25].

### Browse by gene

In this view, multiple alignments of gene loci are shown along with gene structure annotation (exons) and highlighted motif matches (see Figure 5 right). The user is free to scan the genomic region with any motif description as expressed by a IUPAC nucleotide symbol sequence. Surrounding upstream and downstream regions can be considered if desired. A complementary genome browser can be also accessed via the website.

### Browse by motif

A different access point is provided by scanning the whole data set with a user-provided motif description. The conservation level (conserved/not conserved) and scan region (upstream/intronic) can be selected in advance. Two alternative output options either list each individual motif match or summarize motif matches by gene.

## Discussion

We selected a time-course expression profiling experiment of the transition from the dauer state to the non-dauer state and the expression changes after feeding starved L1 animals [26] as an example (see Additional Files 1, 2, 3, 4).

### Feeding of starved L1 animal

At the initial time point (3 hours after inoculation on OP50, Additional File 1), all three pipelines report a weakly similar motif as the initial split point:

TANCCN Kmer pipeline (reverse complement)

AATCNAT GEMODA pipeline

ATHAAT FootPrinter pipeline

The motif that is reported by the GEMODA pipeline is apparently the one that defines the gene set with the most pronounced up-regulation in expression (0.234; set size: n = 88). The conditional tree of the Kmer pipeline reports the motif set, which induces the gene set with the most pronounced down-regulation (-0.1; set size: n = 112).

If we consider the gene expression profile at 6 hours after inoculation (Additional File 2), we first notice the rapid increase of motif candidates that passed the expression significance filter. This increase is conveniently handled by the conditional tree framework, which automatically corrects for multiple testing. All conditional trees pick up motif combinations that are predominantly linked to groups of down-regulated genes.

### Transition from the dauer state to the non-dauer state

For the initial condition (time point 3 hrs, Additional File 3), all three motif discovery pipelines report again a similar first split point:

GCNCTN Kmer pipeline (reverse complement)

GYACTT GEMODA pipeline

GCDCTT FootPrinter pipeline

TGCACT. DAF-12

This sequence resembles the binding site description of DAF-12 [27], a member of the steroid hormone receptor superfamily that affects dauer formation. The set sizes of up-regulated genes carrying these motifs stay the same at a later time point (6 hours, Additional File 4).

The example shows that our motif discovery approach is able to detect known and novel motifs. Hence, we deem it useful for a wide audience of experimentalists.

## Conclusion

We presented an approach to build a motif compendium in *Caenorhabditis *species. To this end, we have computed pairwise alignments of the *Caenorhabditis elegans *genome to three closely related nematode genomes (one finished, one in draft assembly and one newly assembled). The degree of conservation is drastically higher than one would expect from the neutral substitution rate.

From these pairwise alignments we build a multiple alignment and generated alternative motif candidate sets by three different motif discovery strategies. All strategies produce largely overlapping motif candidate lists. That is why, we conclude that the actual motif discovery strategy does have a major effect as long as motifs are evaluated by conservation and expression data.

Our web resource serves as a starting point for biologists to study regulatory elements on a gene by gene basis. Likewise, genome-wide screens for putative gene targets of a particular transcription factor as defined by a consensus motif are easily performed.

Given our set of conserved putative regulatory sequences for the *Elegans *group, it will be exciting to mine for species-specific motif inventions. Phylogenetic profiling on the motif level will be feasible with the advent of more genomes from satellite species (e.g. *Pristionchus pacificus*) and distantly related species (e.g. *Brugia malayi *and *Trichinella spiralis*).

## Availability and requirements

**Project name: **The *Caenorhabditis *Motif Compendium;

**Project home page: **http://corg.eb.tuebingen.mpg.de/CMC;

**Operating system: **Web service running on Linux;

**Programming language: **Perl and R;

**License: **GNU LGPL;

**Any restrictions to use by non-academics: **There are no restrictions on the web site use by non-academics.

## Authors' contributions

CD designed the project and carried out all programming and data analysis. RJS provided conceptual support. CD has written the manuscript. All authors read and approved the final manuscript.

## Supplementary Material

Additional File 1 **Feeding of starved L1 animals – a time course – time point 3 hr**. Starved animals were inoculated onto E. coli seeded plates and grown for 3 hours. **Panel A **shows the conditional tree from the Kmer pipeline. The conditional tree was built from 38 motif candidates. **Panel B **shows the conditional tree from the GEMODA pipeline. The conditional tree was built from 16 motif candidates. **Panel C **shows the conditional tree from the FootPrinter pipeline. The conditional tree was built from 24 motif candidates. Vertices show split point numbers, the motif description and the corresponding P-value of the split (Bonferroni corrected). Edges are labeled with the split conditions. Click here for file

Additional File 2 **Feeding of starved L1 animals – a time course – time point 6 hr**. Starved animals were inoculated onto E. coli seeded plates and grown for 6 hours. **Panel A **shows the conditional tree from the FootPrinter pipeline. **Panel B **shows the conditional tree from the Kmer pipeline. **Panel C **shows the conditional tree from the Gemoda pipeline. All conditional trees were built from 1,000 motif candidates. Vertices show split point numbers, the motif description and the corresponding P-value of the split (Bonferroni corrected). Edges are labeled with the split conditions. Click here for file

Additional File 3 **Transition from the dauer state to the non-dauer state – a time course – time point 3 hr**. Dauers were inoculated onto E. coli seeded plates and grown for 3 hours. **Panel A **shows the conditional tree from the FootPrinter pipeline. **Panel B **shows the conditional tree from the Kmer pipeline. **Panel C **shows the conditional tree from the GEMODA pipeline. Vertices show split point numbers, the motif description and the corresponding P-value of the split (Bonferroni corrected). Edges are labeled with the split conditions. Conditional trees were built from motif candidate sets of size 1,000 (A), 856 (B) and 1,000 (C). Click here for file

Additional File 4 **Transition from the dauer state to the non-dauer state – a time course – time point 6 hr**. Dauers were inoculated onto E. coli seeded plates and grown for 6 hours. **Panel A **shows the conditional tree from the FootPrinter pipeline. **Panel B **shows the conditional tree from the GEMODA pipeline. **Panel C **shows the conditional tree from the Kmer pipeline. Vertices show split point numbers, the motif description and the corresponding P-value of the split (Bonferroni corrected). Edges are labeled with the split conditions. Conditional trees were built from motif candidate sets of size 117 (A), 132 (B) and 475 (C). More supplementary data can be retrieved from [2]. Click here for file
