# Supplementary figures and images for: A Caenorhabditis motif compendium for studying transcriptional gene regulation
Source: BMC Genomics. 2008 Jan 23;9:30. doi: 10.1186/1471-2164-9-30 (PMC2248174; doi:10.1186/1471-2164-9-30)

A

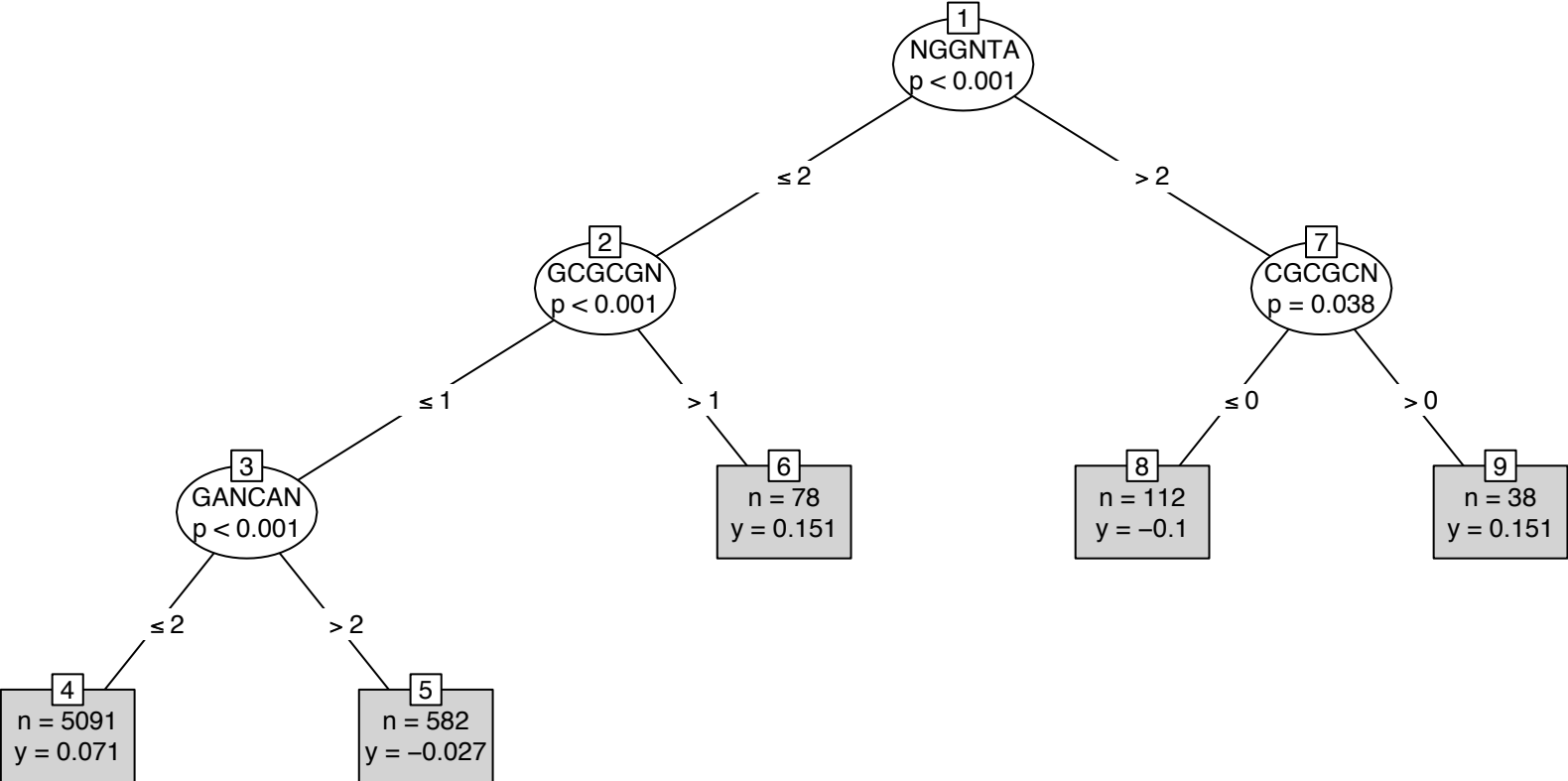

B

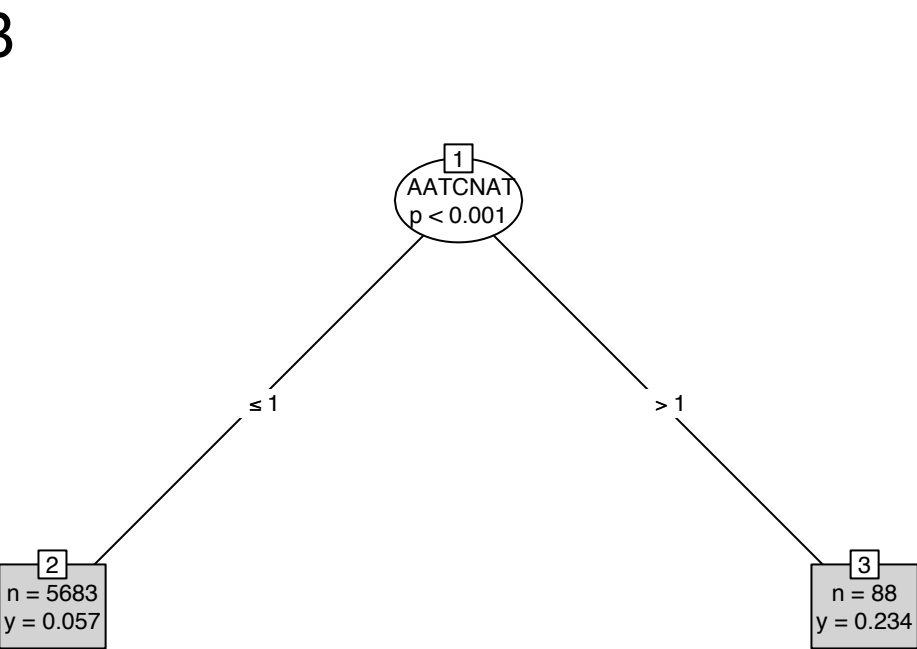

C

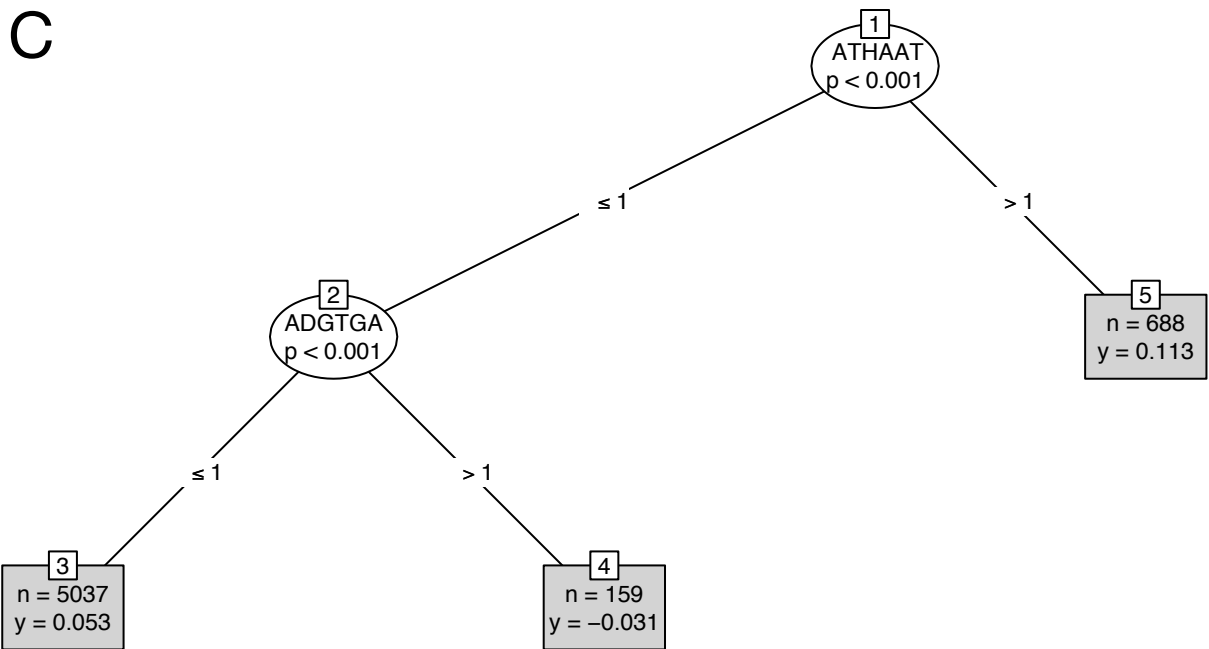

Supplement: Additional File 1 — Feeding of starved L1 animals – a time course – time point 3 hr. Starved animals were inoculated onto E. coli seeded plates and grown for 3 hours. Panel A shows the conditional tree from the Kmer pipeline. The conditional tree was built from 38 motif candidates. Panel B shows the conditional tree from the GEMODA pipeline. The conditional tree was built from 16 motif candidates. Panel C shows the conditional tree from the FootPrinter pipeline. The conditional tree was built from 24 motif candidates. Vertices show split point numbers, the motif description and the corresponding P-value of the split (Bonferroni corrected). Edges are labeled with the split conditions. [file 1471-2164-9-30-S1.PDF]

A

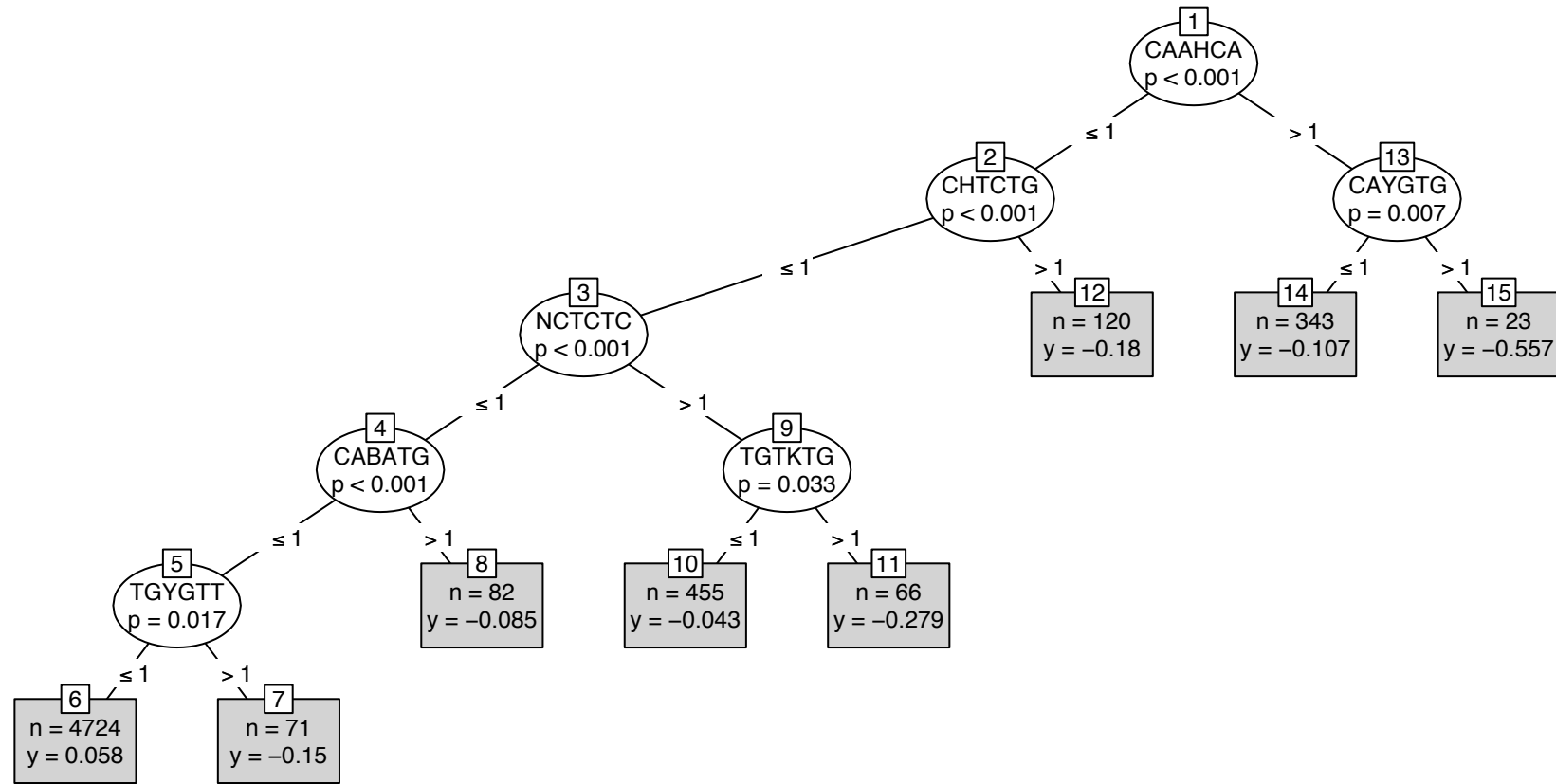

B

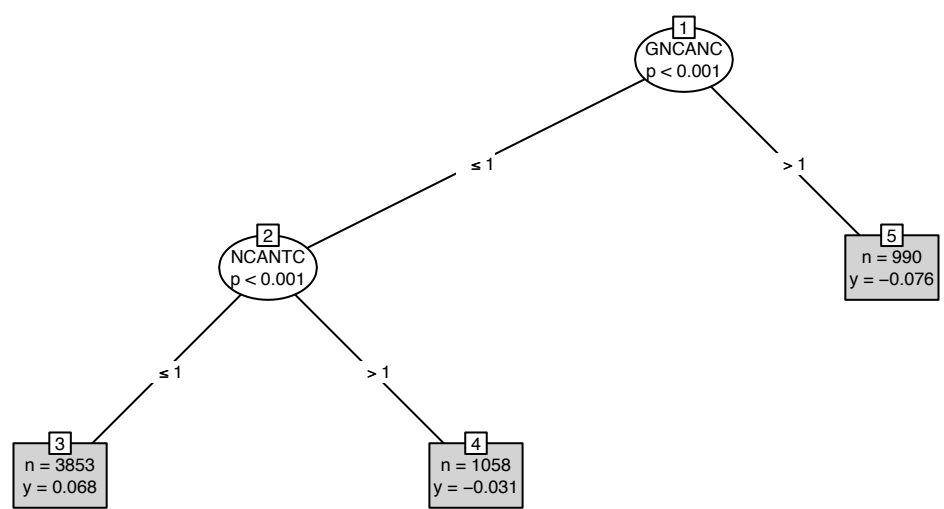

C

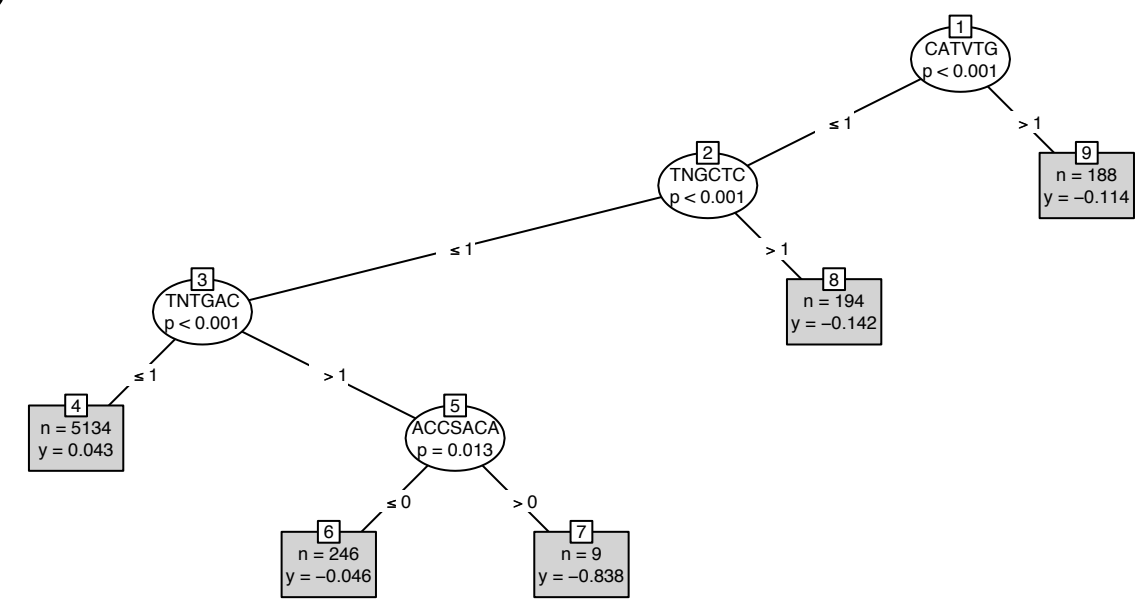

Supplement: Additional File 2 — Feeding of starved L1 animals – a time course – time point 6 hr. Starved animals were inoculated onto E. coli seeded plates and grown for 6 hours. Panel A shows the conditional tree from the FootPrinter pipeline. Panel B shows the conditional tree from the Kmer pipeline. Panel C shows the conditional tree from the Gemoda pipeline. All conditional trees were built from 1,000 motif candidates. Vertices show split point numbers, the motif description and the corresponding P-value of the split (Bonferroni corrected). Edges are labeled with the split conditions. [file 1471-2164-9-30-S2.PDF]

A

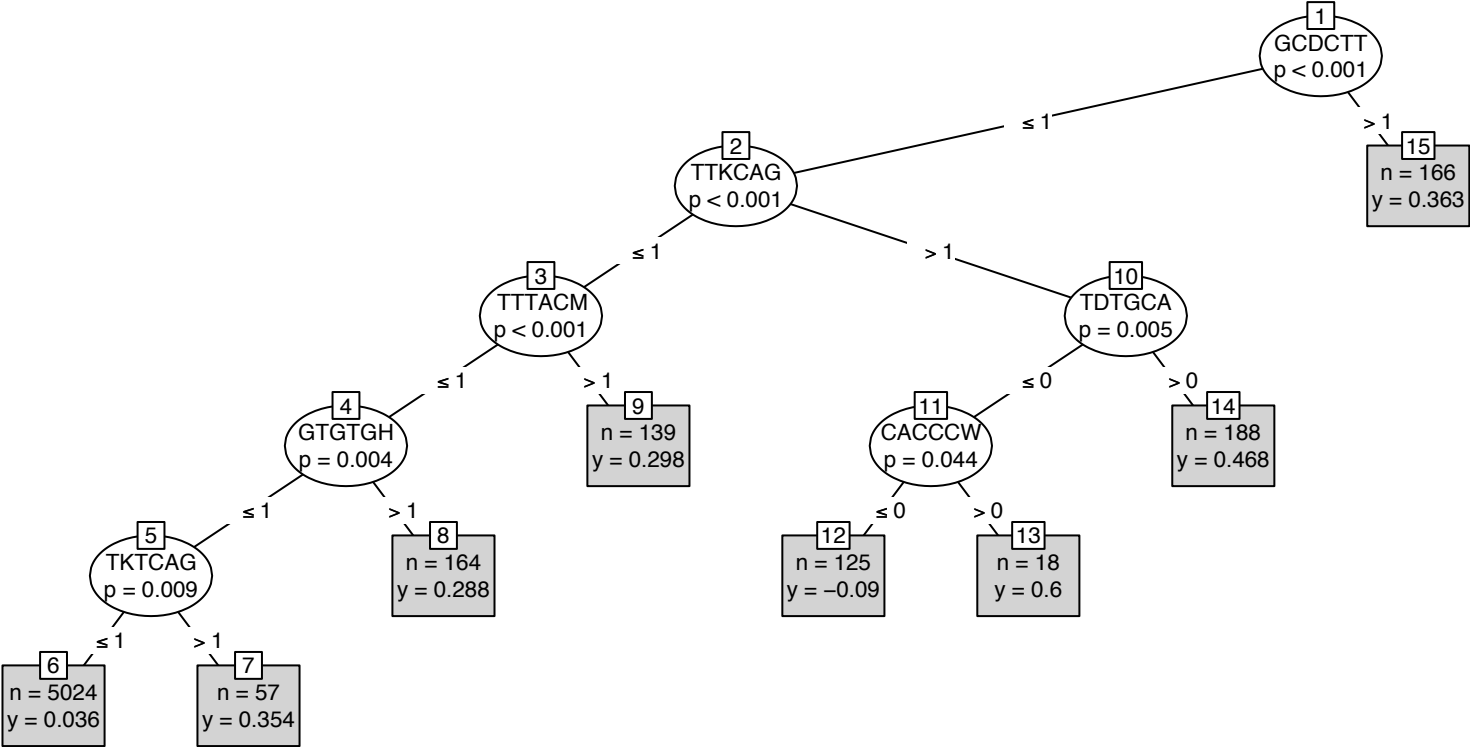

B

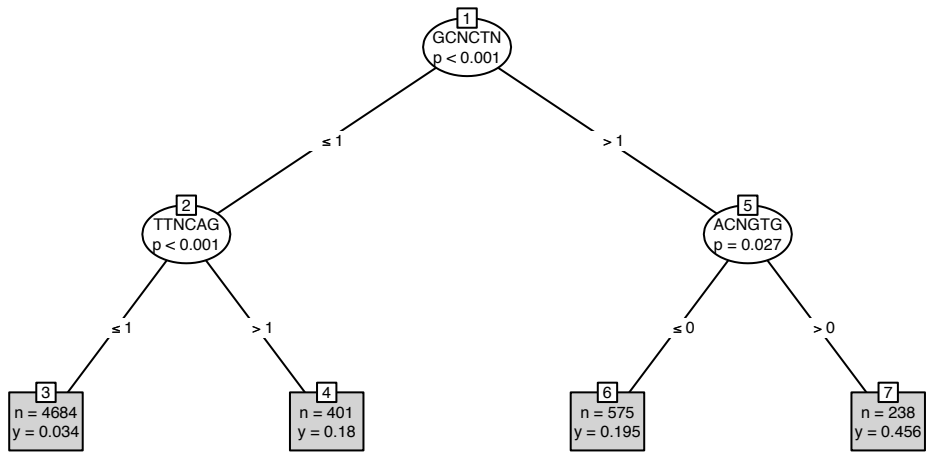

C

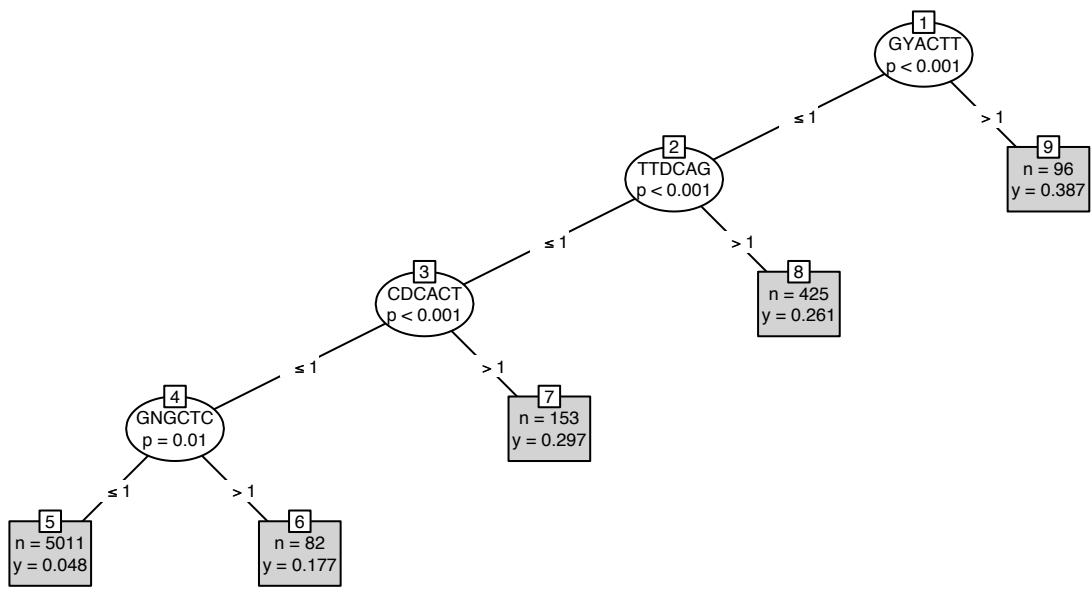

Supplement: Additional File 3 — Transition from the dauer state to the non-dauer state – a time course – time point 3 hr. Dauers were inoculated onto E. coli seeded plates and grown for 3 hours. Panel A shows the conditional tree from the FootPrinter pipeline. Panel B shows the conditional tree from the Kmer pipeline. Panel C shows the conditional tree from the GEMODA pipeline. Vertices show split point numbers, the motif description and the corresponding P-value of the split (Bonferroni corrected). Edges are labeled with the split conditions. Conditional trees were built from motif candidate sets of size 1,000 (A), 856 (B) and 1,000 (C). [file 1471-2164-9-30-S3.PDF]

A

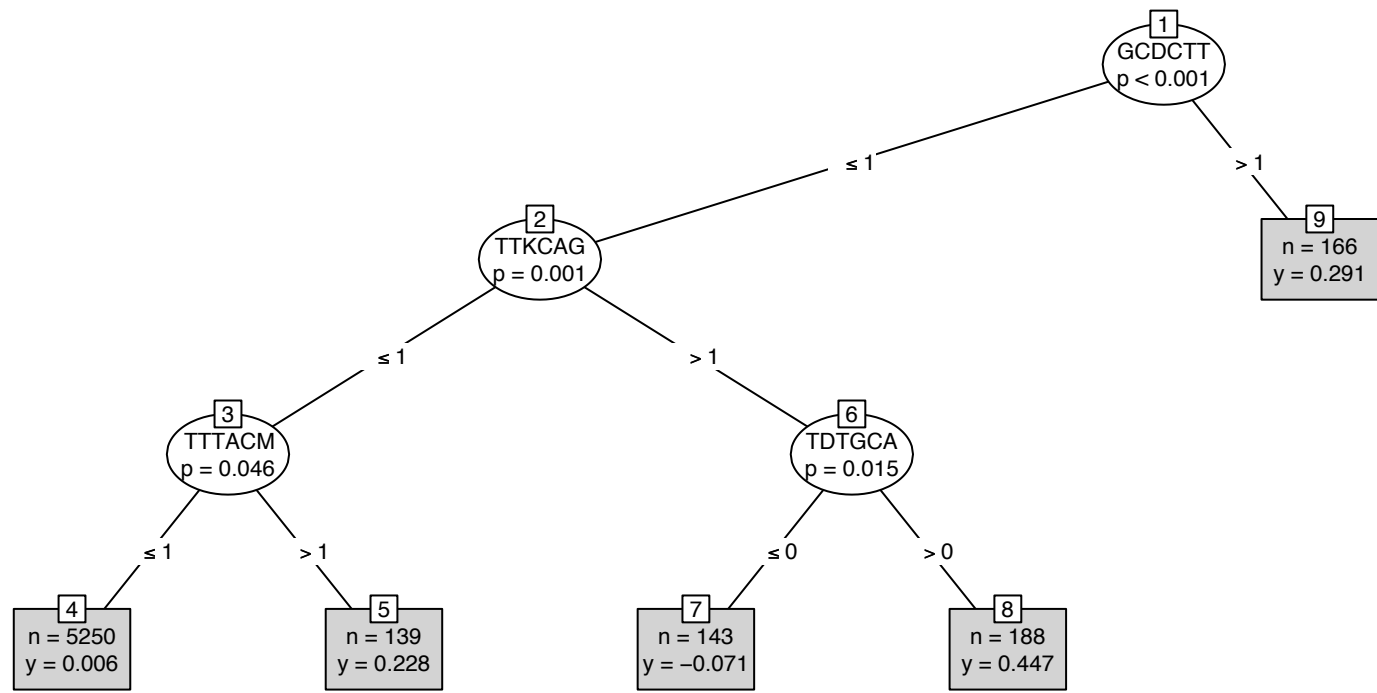

B

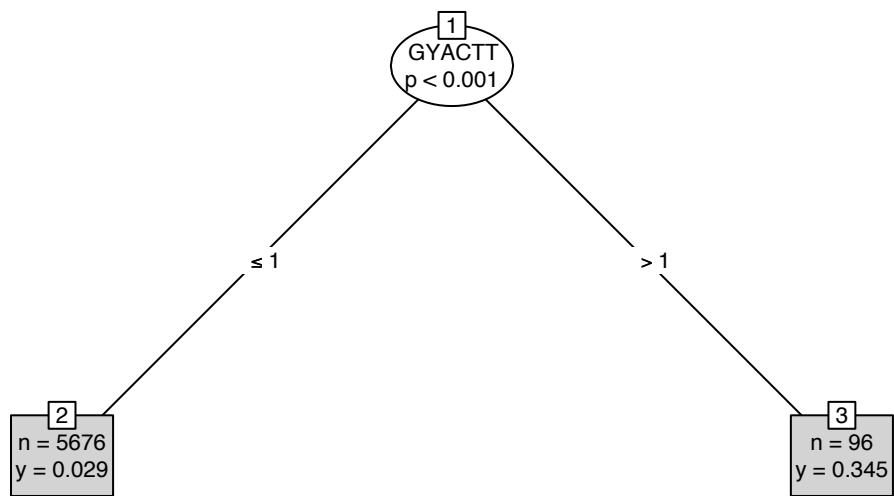

C

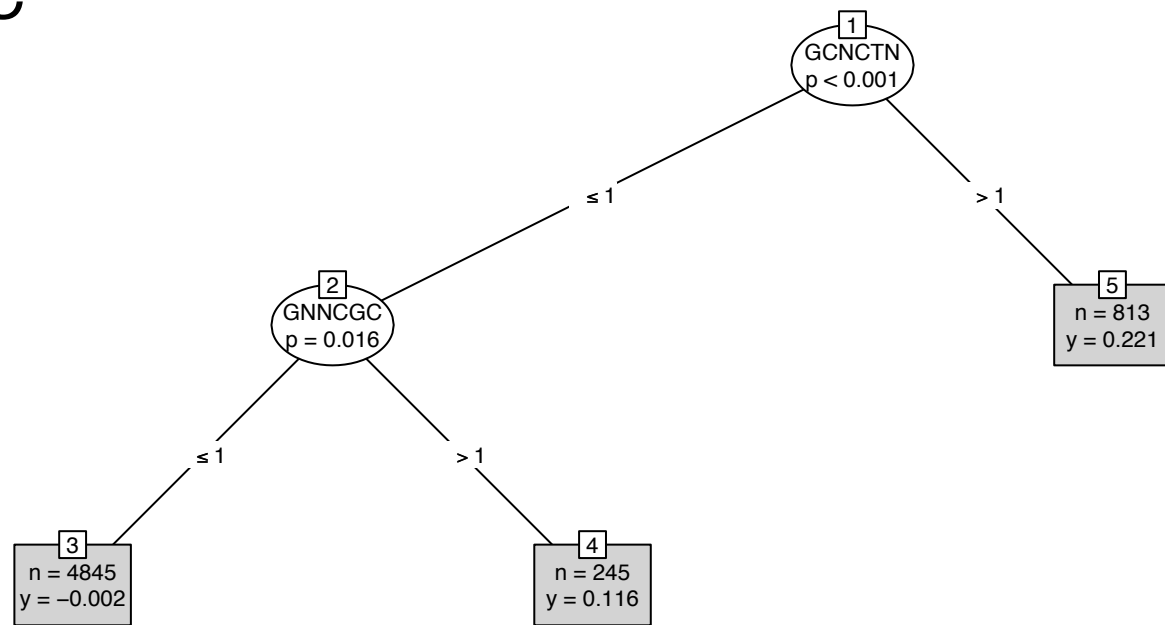

Supplement: Additional File 4 — Transition from the dauer state to the non-dauer state – a time course – time point 6 hr. Dauers were inoculated onto E. coli seeded plates and grown for 6 hours. Panel A shows the conditional tree from the FootPrinter pipeline. Panel B shows the conditional tree from the GEMODA pipeline. Panel C shows the conditional tree from the Kmer pipeline. Vertices show split point numbers, the motif description and the corresponding P-value of the split (Bonferroni corrected). Edges are labeled with the split conditions. Conditional trees were built from motif candidate sets of size 117 (A), 132 (B) and 475 (C). More supplementary data can be retrieved from [2]. [file 1471-2164-9-30-S4.PDF]
